# Supplementary figures and images for: Nrf2 signaling promotes cancer stemness, migration, and expression of ABC transporter genes in sorafenib-resistant hepatocellular carcinoma cells
Source: PLoS One. 2021 Sep 2;16(9):e0256755. doi: 10.1371/journal.pone.0256755 (PMC8412368; doi:10.1371/journal.pone.0256755)

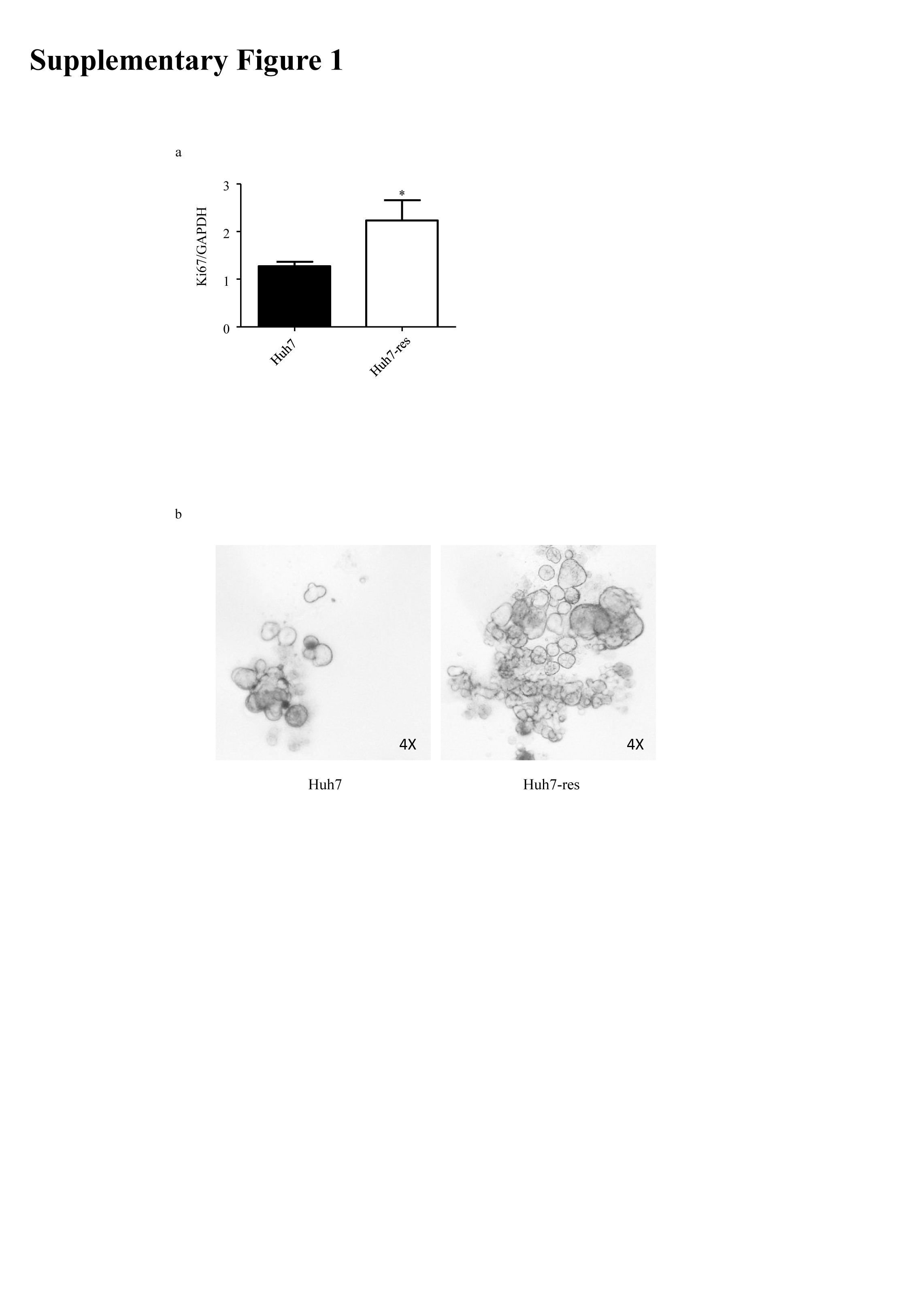

Supplement: S1 Fig — a. PCR quantification of Ki67 mRNA expressed in Huh7 cells and sorafenib resistant Huh7 cells, expressed for 48h cultured in DMEM (10%FBS). (n = 4) * indicates P value<0.05) ** indicates P value<0.01) *** indicates P value<0.001. b. Sphere forming assay was performed in Huh7 cells and sorafenib resistant Huh7 cells, expressed for 7 days cultured in sphere culture medium. (JPG) [file pone.0256755.s001.jpg]

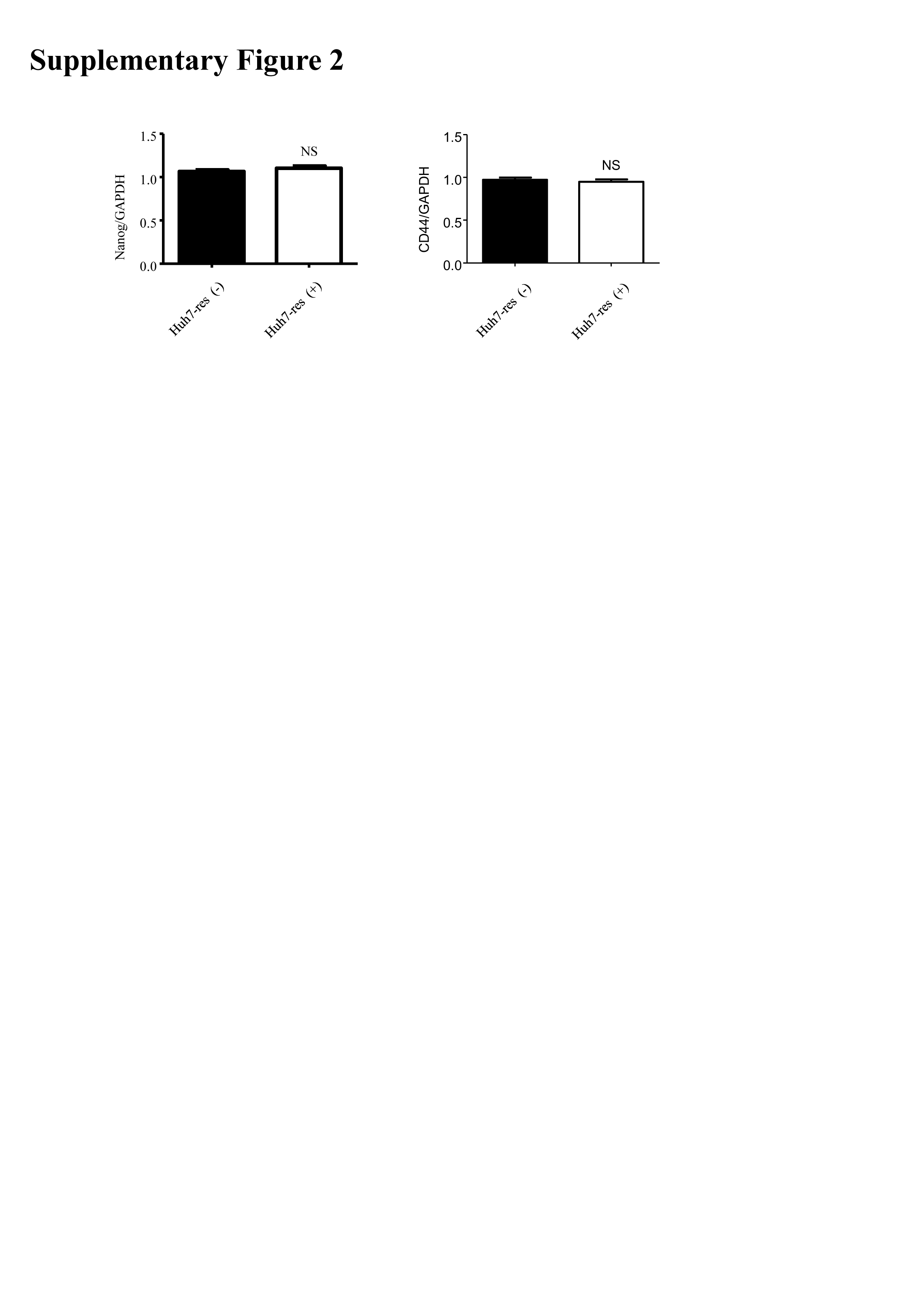

Supplement: S2 Fig — PCR quantification of Nanog and CD44 mRNA expressed in Huh7-res cells, expressed for 48h cultured in DMEM (10%FBS) with the presence or absence of sorafenib. (n = 4) NS indicates P value≥0.05. (JPG) [file pone.0256755.s002.jpg]

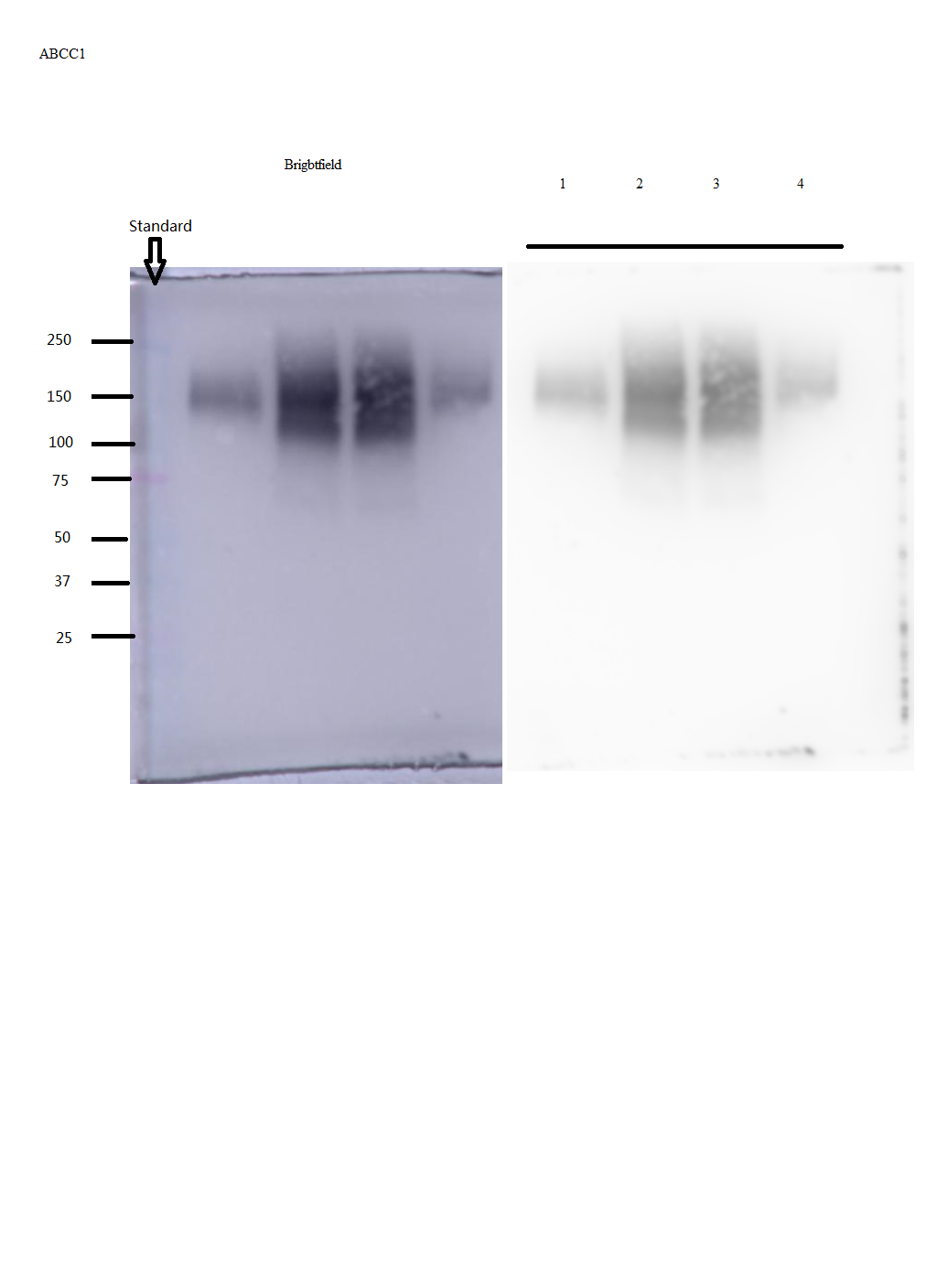

Supplement: S3 Fig — (TIF) [file pone.0256755.s003.tif]

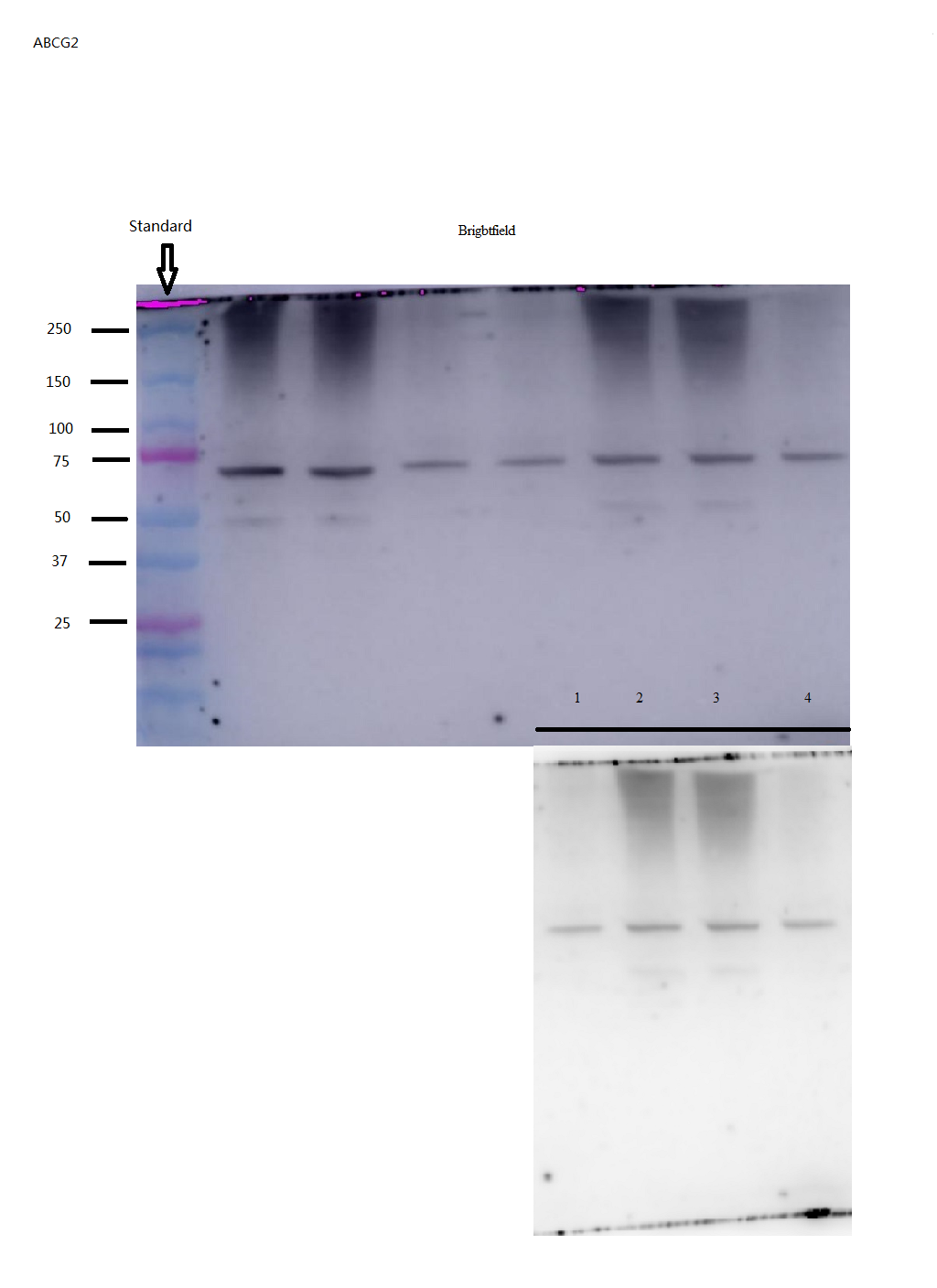

Supplement: S4 Fig — (TIF) [file pone.0256755.s004.tif]

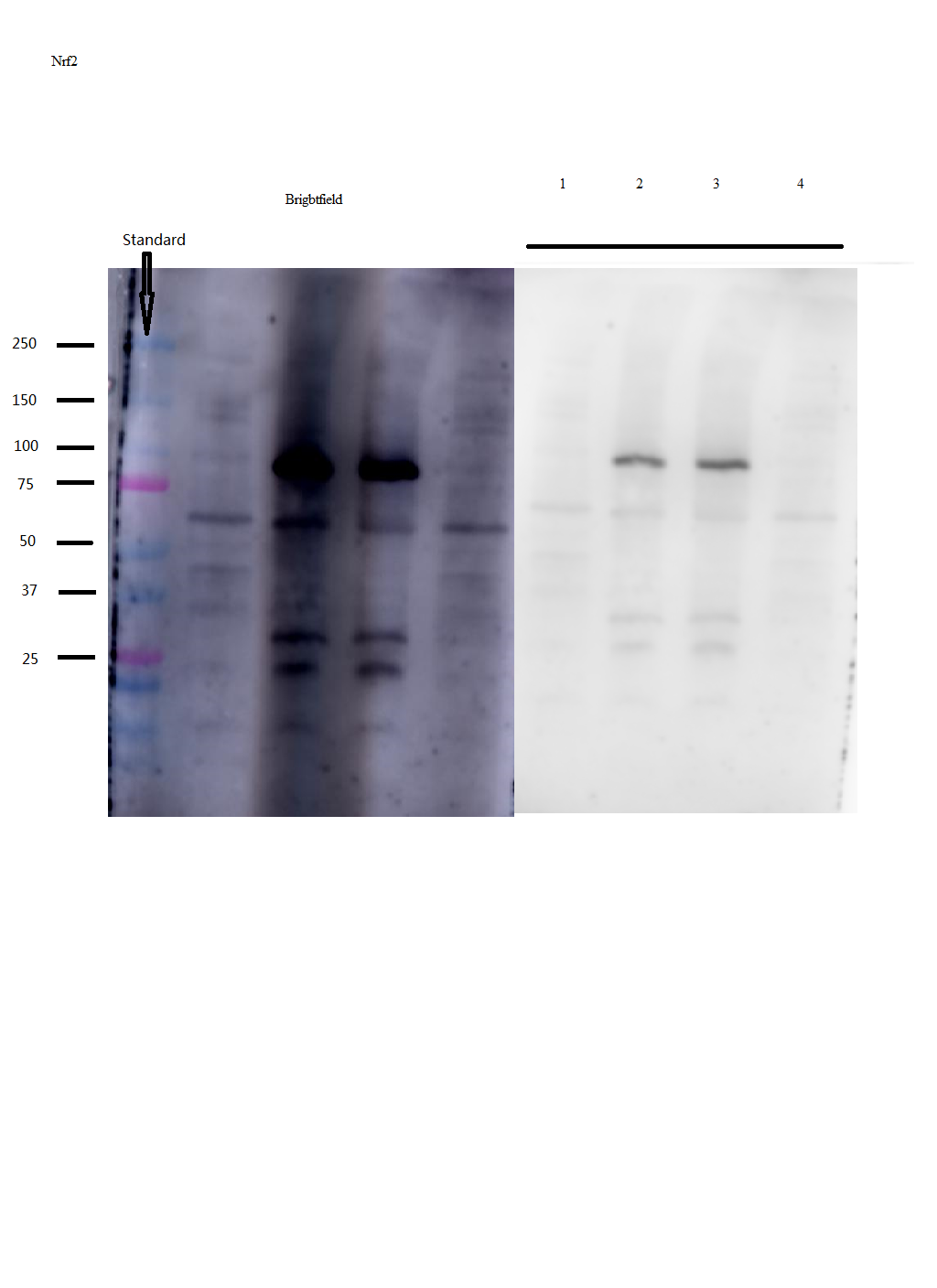

Supplement: S5 Fig — (TIF) [file pone.0256755.s005.tif]

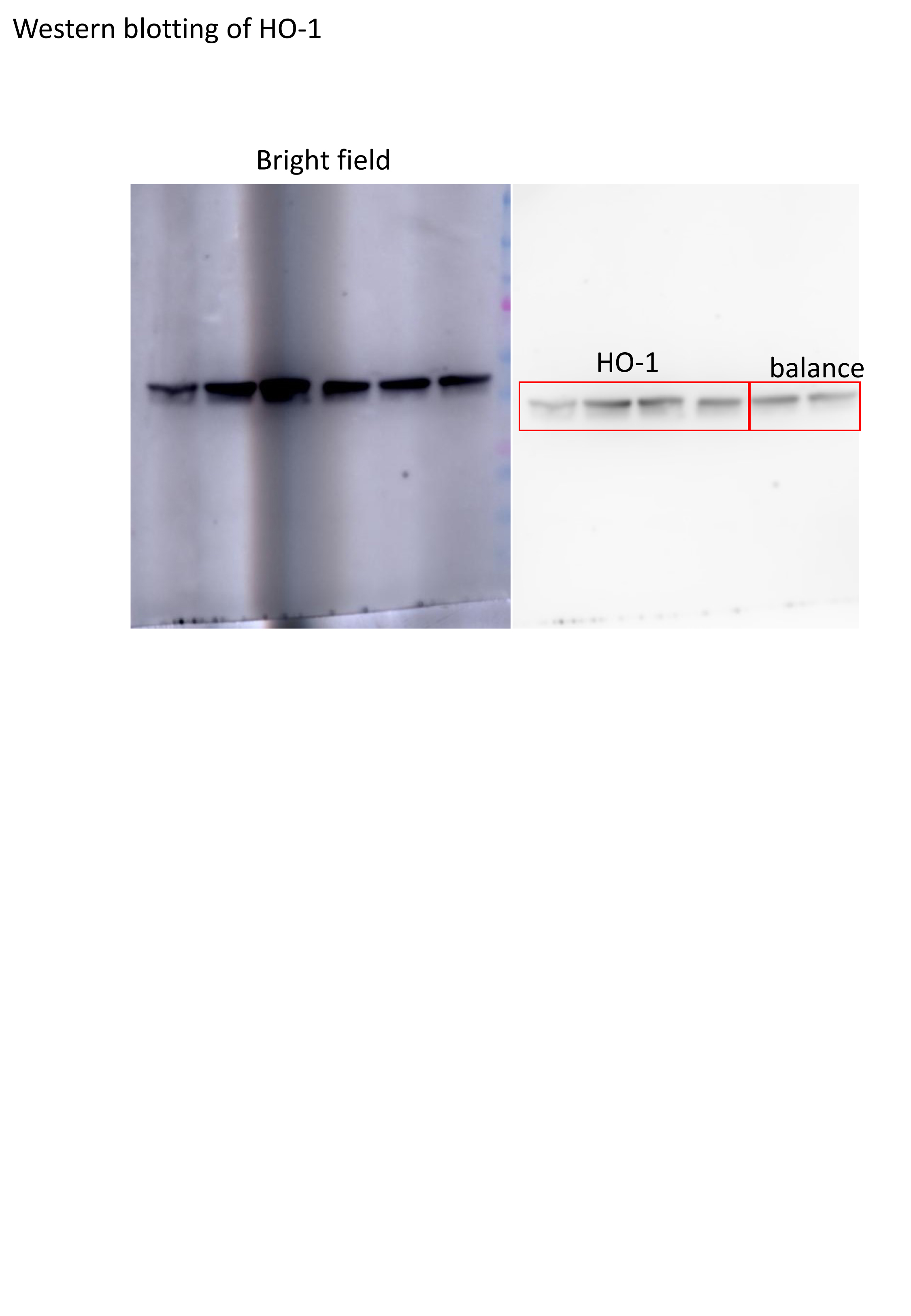

Supplement: S6 Fig — (JPG) [file pone.0256755.s006.jpg]
