# Supplementary material for: Nrf2 signaling promotes cancer stemness, migration, and expression of ABC transporter genes in sorafenib-resistant hepatocellular carcinoma cells
Source: PLoS One. 2021 Sep 2;16(9):e0256755. doi: 10.1371/journal.pone.0256755 (PMC8412368; doi:10.1371/journal.pone.0256755)
Supplement: S1 Table — (DOCX) [file pone.0256755.s007.docx]

**Supplementary Table 1.** Primer sequence of gene used for qPCR.

| **Gene name**  *Human* | **Primer sequence** |
| --- | --- |
| ABCA6 | F: AAACAGAAAAGCGTGTATCAGCA R: AAACAGAAAAGCGTGTATCAGCA |
| ABCB1 | F: TTGGCTGATGTTTGTGGGAAG R: CCAAAAATGAGTAGCACGCCT |
| ABCC1 | F: GTGAATCGTGGCATCGACATA R: GCTTGGGACGGAAGGGAATC |
| ABCG2 | F: CAGGTGGAGGCAAATCTTCGT R: ACCCTGTTAATCCGTTCGTTTT |
| CD44 | F: CTGCCGCTTTGCAGGTGTA  R: CATTGTGGGCAAGGTGCTATT |
| EPCAM | F: AATCGTCAATGCCAGTGTACTT R: AATCGTCAATGCCAGTGTACTT |
| GAPDH | F: TGTGGGCATCAATGGATTTGG R: ACACCATGTATTCCGGGTCAAT |
| HMOX1 (HO-1) | F: AAGACTGCGTTCCTGCTCAAC R: AAAGCCCTACAGCAACTGTCG |
| Nanog | F: TCCCGAGAAAAGATTAGTCAGCA R: AGTGGGGCACCTGTTTAACTT |
| Nrf2 | F: GGCGTTAGAAAGCATCCTTCC R: GCAGAGGGCACACTCAAAGT |
