# Supplementary material for: Nrf2 signaling promotes cancer stemness, migration, and expression of ABC transporter genes in sorafenib-resistant hepatocellular carcinoma cells
Source: PLoS One. 2021 Sep 2;16(9):e0256755. doi: 10.1371/journal.pone.0256755 (PMC8412368; doi:10.1371/journal.pone.0256755)
Supplement: Attachment — Submitted filename: Response to reviewers.docx [file pone.0256755.s008.docx]

**PONE-D-20-37563
Nrf2 signaling promotes cancer stemness, migration, and expression of ABC transporter genes in sorafenib-resistant hepatocellular carcinoma cells
PLOS ONE**

**Point by point responses to the referees and the editors**

First of all, thank you so much for your kind consideration of our manuscript. Also, I appreciate your and reviewers’ valuable comments to improve quality of our manuscript. According to the associate editor’s and reviewers’ comments, the manuscript has been fully revised.

Revised points responding to the reviewers’ comments are described below.

Reviewer #1: The authors have shown that Nrf2 signaling promotes cancer stemness, migration, and expression of ABC transporter genes in sorafenib-resistant hepatocellular carcinoma cells. The work is interesting, however there are several drawbacks.

Major concern:

1. Introduction portion is not well written and needs to be improved.

We appreciate the helpful comment. We revised the introduction part according to the guideline provided by Plos One.

2. You have given references related to KEAP1/ NRF2 pathway and the role of CSCs in cancer recurrence and chemotherapy resistance in the result section. These should be mentioned in the introduction section.

We appreciate the valuable comment. We add the introduction of relationship between Nrf2 and chemotherapy resistance in cancer stem cells as below. The dark side of Nrf2 signaling was also described in cancer stem cells. Activated Nrf2 resulted in less ROS production and refractory response to drugs [1]. Nrf2, as a transcription factor, contributed to tumor generation of cancer stem cells by gene editing technics [2].

2. Figure quality is poor. I will suggest you to provide better figures for wound healing assays (Figure 2 and 6).

Thank you for your valuable suggestions. Figures 2 and 6 have been revised to improve the quality.

3. All the conclusions are based only on RT-PCR data. You should provide western blot data for further validation.

We appreciate the helpful comment. We performed western blotting to detect the protein level of Nrf2, HO-1, ABCC1 and ABCG2 in Huh7, sorafenib-resistant Huh7, and Nrf2 knocked down sorafenib-resistant Huh7 cells. The results were shown in figure 5c. And we additionally described the following sentence in result section; The expression in protein level of Nrf2, HO-1, ABCC1 and ABCG2 showed the similar results (Fig.5c).

Minor mistake:

1. What do you mean by “ideal” confluency in the wound healing assay?

Thank you for your valuable comment. In our study, the ”ideal” confluency means adherent cells reach 90% of the area. I am sorry that we used confusing description, so we changed the sentence as below; Parental and sorafenib-resistant Huh7 cells were plated in 6 well plates. Then confluent cells were scratched with a 10 μL pipette tip to create a ~0.5-mm-wide gap through the entirety of each cell layer.

2. The sequence of siRNA and primers are missing.

We appreciate the helpful comment. The primer sequences used in this study are provided in Supplementary Table 1. The Nrf2 siRNA (s9492) was purchased from Applied Biosystems, Waltham Massachusetts USA. These information were also added in Materials and methods section.

3. Mention the amount of total RNA used in cDNA synthesis.

We appreciated the useful suggestion. We used 2.5μg total RNA for every sample in cDNA synthesis. Now we have added this in Materials and methods section.

4. Please provide the PCR protocol in details.

Thank you for your advice. We added details of PCR protocol in Materials and methods section.

Reviewer #2: In this manuscript, Gao et al. demonstrate that cancer stemness, migration/invasion and expression of ABC transporter genes are upregulated in the sorafenib-resistant hepatocellular carcinoma cells (HCC) they generated. Knockdown of Nrf2 reduced cancer stemness, migration/invasion and expression of ABC transporter genes in the sorafenib-resistant HCC cells. The authors propose that targeting Nrf2 signaling pathway has therapeutic potential in sorafenib-resistant HCC. This is an interesting work and it has been shown overexpression of Nrf2 in HCCs and Nrf2 regulates oxidative stress response and drives tumorigenesis. However, some additional experiments and modifications/data interpretation to the manuscript should be addressed to strengthen the discovery.

Fig.1A Is the difference of cell survival rate between Huh7 and Huh7-res cells statistically significant?

We appreciate the helpful comment. To compare the difference of IC50 between 2 groups, we repeated the cell viability assay in two cell lines at the same time according to reported protocols [4]. We cultured Huh7 and sorafenib-resistant Huh7 in 96 well flat bottom plate by 2x104 cells/well. Cells were co-cultured with increasing dose of sorafenib for 48 hours, then incubated in 10% cell-counting kit-8 culture medium for 1-4 hours. (n=4) Absorbance at 450 nm was then measured with a plate reader. IC50 of sorafenib was calculated using GraphPad Prism 5 software. Results showed that IC50 of Huh7 was 10.68μM, IC50 of sorafenib resistant Huh7 was 16.48μM. Two way ANOVA was performed to evaluate the difference between two groups. P value was less than 0.001. The difference of cell survival rate between Huh7 and Huh7-res cells was statistically significant. We described it in the result section as follows; Results showed that IC50 of Huh7 was 10.68μM, IC50 of sorafenib resistant Huh7 was 16.48μM（P value<0.001）.

Fig.1B Methodological detail of Fig. 1B is missing. Furthermore, no precise measurement of the difference of cell volume or cytoplasm between Huh7 and Huh7-res cells and statistical analysis. In addition, please indicate vacuole structures.

Thank you for your valuable comment. We described the methods of HE staining in the Method section. The measurement of cell volume was performed by ImageJ. Then we described it in Results section as follows; Compared with parental Huh7 cells, the sorafenib-resistant Huh7 cells displayed more heterogeneous morphology; the cell volume increased, the cytoplasm and vacuole structures were visible. We added markers（△） to point out vacuole structures.

Fig. 2A&B Fig2C showed higher proliferation of Huh7-res cells vs. Huh7 cell in the presence of sorafenib in a 3-week assay. Are there any proliferation data in short time period, with and without sorafenib? Does the proliferation difference affect the results of migration and wound healing assay in 24h?

We appreciated the useful suggestion. Firstly, we added RT-PCR result of ki67 in Huh7 and resistant Huh7 cells, both cultured in DMEM (10%FBS) for 48 hours. The result was added in Supplement Figure 1a. Secondly, when we performed migration and wound healing assay, we used serum-free medium, so that the proliferation difference did not affect the invasion and migration results.

Fig.3 What are the mRNA levels of EPCAM, Nanog, CD44 in tumor sphere assay, a three-dimensional culture setting between Huh-7 and Huh-res cells? In addition, this experiment lacks methodological detail for other researcher to repeat the experiment, what is the PCR primer set for EPCAM, Nanog and CD44. Another question is what are the mRNA levels of EPCAM, Nanog, CD44 in the presence or absence of sorafenib in Huh7-res cells?

Thank you for your kind advise. Firstly, we added the tumor sphere forming assay in supplementary figure 1b [5-7]. The results showed that stemness in resistant cells was significantly higher. Then, the PCR primers’ sequences have been described in supplementary table 1. Lastly, we compared gene expression of stemness markers in the presence or absence of sorafenib in Huh7-res cells. As the resistant cell line was developed by dose increasing treatment and stably cultured in high dose of sorafenib for over 3 months, the results showed that a short-time absence of sorafenib did not make statistic difference.

Fig. 4. What are the protein levels of Nrf2, HO-1, ABCC1 ABCG2 and ABCB1 and ABCA6? Western blot showing protein levels of these proteins will strengthen this part of the paper.

We appreciate the helpful comment. As transporters ABCC and ABCG were most commonly regarded with sorafenib therapy [3], and in current situation, it takes too much time to purchase other two kinds of ABC transporters, we performed western blotting to detect the protein level of Nrf2, HO-1, ABCC1 and ABCG2 in Huh7, sorafenib-resistant Huh7, and Nrf2 knocked down sorafenib-resistant Huh7 cells. The results were showed in figure 5c.

Fig. 5 A non-targeting siRNA control is not included, but that needs to demonstrate the reduction of Nrf2, CD44, Nanog was not because of transfection itself.

We appreciate the valuable comment. We added siRNA negative as a control and modified the figures. We showed the results in Figure 5a-b. And we added the description in the Materials and Methods section as well as Result section.

Fig.6 Similar to the point above, how cell proliferation was affected by knockdown Nrf2? Again, is the invasion or migration measured here because of cell proliferation?

Thank you for your useful advice. We added colony forming assay in resistant Huh7 and Nrf2 knocked down cells. The results were showed in Figure 6c. During the invasion or migration assay, cells were cultured in serum free medium. As the above explanation, the proliferation difference did not affect the invasion and migration results.

**Additional references**

1. Choi, Bo-Hyun, Jin Myung Kim, and Mi-Kyoung Kwak. "The multifaceted role of NRF2 in cancer progression and cancer stem cells maintenance." Archives of Pharmacal Research (2021): 1-18.

2. Bi, Zhuoyue, et al. "Cooperation between NRF2-mediated transcription and MDIG-dependent epigenetic modifications in arsenic-induced carcinogenesis and cancer stem cells." Seminars in Cancer Biology. Academic Press, 2021.

3. Wang, Yi-Jun, et al. "Repositioning of tyrosine kinase inhibitors as antagonists of ATP-binding cassette transporters in anticancer drug resistance." Cancers 6.4 (2014): 1925-1952.

4. Zhao, Zhongwei, et al. "Sophoridine suppresses lenvatinib‐resistant hepatocellular carcinoma growth by inhibiting RAS/MEK/ERK axis via decreasing VEGFR2 expression." Journal of Cellular and Molecular Medicine 25.1 (2021): 549-560.

5. Ma, Xiao-Lu, et al. "CD73 sustained cancer-stem-cell traits by promoting SOX9 expression and stability in hepatocellular carcinoma." Journal of hematology & oncology 13.1 (2020): 1-16.

6. Ma, Xiao-Lu, et al. "Sphere-forming culture enriches liver cancer stem cells and reveals Stearoyl-CoA desaturase 1 as a potential therapeutic target." BMC cancer 19.1 (2019): 1-12.

7. Yamashita, Taro, et al. "EpCAM-positive hepatocellular carcinoma cells are tumor-initiating cells with stem/progenitor cell features." Gastroenterology 136.3 (2009): 1012-1024.
