# Supplementary material for: Nrf2 signaling promotes cancer stemness, migration, and expression of ABC transporter genes in sorafenib-resistant hepatocellular carcinoma cells
Source: PLoS One. 2021 Sep 2;16(9):e0256755. doi: 10.1371/journal.pone.0256755 (PMC8412368; doi:10.1371/journal.pone.0256755)
Supplement: Attachment — Submitted filename: renamed_5a7ce.docx [file pone.0256755.s009.docx]

**PONE-D-20-37563R1
Nrf2 signaling promotes cancer stemness, migration, and expression of ABC transporter genes in sorafenib-resistant hepatocellular carcinoma cells
PLOS ONE**

**Point by point responses to the referees and the editors**

First of all, thank you so much for your kind consideration of our manuscript. Also, I appreciate your and reviewers’ valuable comments to improve quality of our manuscript. According to the associate editor’s and reviewers’ comments, the manuscript has been fully revised.

Revised points responding to the reviewers’ comments are described below.

**Reviewer #1: The manuscript has been improved significantly. However, you have to take care of a few things to improve it further.**

**1. The figures of wound healing/ migration assays are still not clear.**

We appreciate the valuable comment. We repeated the experiments and modified the Figure 2b, 6b.

**2. The HO-1 western blot image is not good.**

Thank you for your valuable comment. We repeated the western blotting of HO-1 expression, the result was showed in Figure 5c.

**3. The fonts used in the figures are hazy. Please try to make it clearly visible. The supplementary figures' fonts are better than the fonts used in the main figures of the manuscript.**

We appreciated the useful suggestion. We modified the fonts in all figures.

**4. Please use the same font type throughout your manuscript.**

We appreciate the helpful comment. The font type of the manuscript has been modified.

**5. Some typos are there in the manuscript, for eg,
page 58, line 3: “Nrf2 helps maintain the oxidative stress balance”
page 63: “asaay”
page 64: “Nuceis”**

Thank you very much. I’m sorry we used some misspelled description, they have been corrected.

**Reviewer #2: The authors have clearly responded to my questions and made the necessary changes. I would like to recommend the revised version of the manuscript for publication. Minor points/comments regarding the figures:

Fig. 1a In the text it states: The IC50 of Huh7 was 10.68μM, IC50 of sorafenib resistant Huh7 was 16.48μM; however, in the figure the x axis label for Sorafenib log concentration is nM.**

We appreciate the helpful comment. We corrected the mistake.

**Fig. 2a right panel missing "-" in Sorafenib treatment line.**

Thank you for your valuable comment. The figure has been corrected.

**Fig. 6b: the quality of the figure needs improvement.**

Thank you for your advice. We repeated the experiment and modified the Figure 6b.
